# Reduced orthodontic tooth movement in *Ank* knockout mice

**DOI:** 10.1093/jbmrpl/ziaf064

**Published:** 2025-04-17

**Authors:** Marta Rizk, Emily Yin Chu, Rogerio Bastos Craveiro, Merve Elmas, Sihem Brenji, Christian Niederau, Nikolaus Marx, Brian Lee Foster, Martha Joan Somerman, Michael Wolf

**Affiliations:** Department of Orthodontics, University Hospital Aachen, Aachen 52074, Germany; Department of Biomaterials and Regenerative Dental Medicine, University of Maryland School of Dentistry, Baltimore, MD 21201-1786, United States; Department of Orthodontics, University Hospital Aachen, Aachen 52074, Germany; Department of Orthodontics, University Hospital Aachen, Aachen 52074, Germany; Department of Orthodontics, University Hospital Aachen, Aachen 52074, Germany; Department of Orthodontics, University Hospital Aachen, Aachen 52074, Germany; Department of Internal Medicine I—Cardiology, Angiology and Intensive Care Medicine, University Hospital RWTH Aachen, Aachen 52074, Germany; Division of Biosciences, College of Dentistry, The Ohio State University, Columbus, OH 43210-1267, United States; National Institute of Arthritis and Musculoskeletal and Skin Disease, National Institutes of Health, Bethesda, MD 20892-3675, United States; Department of Orthodontics, University Hospital Aachen, Aachen 52074, Germany

**Keywords:** bone QCT/microCT, dental biology, bone histomorphometry, disorders of calcium/phosphate metabolism, bone modeling and remodeling

## Abstract

Hypercementosis has been previously reported in mice lacking progressive ankylosis protein (*Ank* KO mice, or *Ank, KO - knockout, WT - wildtype*) due to decreased levels of the mineralization inhibitor inorganic pyrophosphate. However, the impact of hypercementosis on alveolar bone remodeling and periodontal ligament (PDL) maintenance from orthodontic forces during orthodontic tooth movement (OTM) remains unclear. To investigate the roles of ANK protein on tooth movement, PDL maintenance, alveolar bone remodeling, and tooth root resorption, we performed a split-mouth model of OTM induced by a closed-coil spring stretched between the maxillary first molar and maxillary incisors in *Ank* KO and WT mice (including both males and females). Micro-computed tomographic analysis revealed a 36.6% reduction in OTM in *Ank* KO mice compared with WT mice, although OTM-induced thickening of PDL was found to be similar in both groups. While reduced tissue mineral density (TMD) of the alveolar bone was observed in WT mice, TMD in *Ank* KO mice was maintained. Loss of *Ank* leads to wider roots with thicker cementum on the untreated, contralateral side, whereas a significant increase in OTM-induced root resorption was observed on the lateral tension side. Histologic analysis of root resorption confirmed these data and showed increased resorption lacunae located prevalently in the OTM tooth root cementum of *Ank* KO mice. Using a quantitative PCR array of bone-associated markers to interrogate total RNA harvested from PDL tissues along the root surface, we found alterations in gene expression from OTM in both WT and *Ank* KO mice, which included genes involved in bone remodeling, calciotropic hormones and receptors, cytokines, growth factors, and receptors. Our findings advance the understanding of the role of *Ank* in regulating mineralization in the periodontium as well as factors involved in root resorption.

## Introduction

Orthodontic tooth movement (OTM) induces remodeling of periodontal tissues, including alveolar bone and periodontal ligament, and occasionally root cementum. Orthodontic tooth repositioning results from alveolar bone formation and resorption processes induced by mechanical stimulation on the tension and compression sides of tooth roots. Dysregulation of these processes may result in degeneration of alveolar bone, cementum, and, in severe cases, the underlying dentin. Tooth root resorption is a significant side effect of orthodontic treatment that often becomes irreversible when extended into dentin. Cementum is thought to act as a protective barrier against osteoclast and odontoclast activity during such inflammatory processes.^1^ Deeper insight into the molecular regulators of cementogenesis and periodontal tissue homeostasis, and crosstalk between periodontal tissues, may inform strategies to prevent root resorption and periodontal degradation.^2^

Cementum formation is regulated by inorganic pyrophosphate (PP_i_) levels. Extracellular PP_i_ concentrations are modulated by 3 primary proteins. Tissue-nonspecific alkaline phosphatase (gene: *Alpl*; protein; TNAP) is a cell surface enzyme responsible for the hydrolysis of PP_i_ to generate inorganic phosphate (P_i_).^3^ The progressive ankylosis protein (*Ank/*ANKH; ANK) was found to regulate transport of PP_i_ or adenosine triphosphate (ATP) to extracellular spaces,^4^^,^^5^ although the direct mechanism of PP_i_ regulation through ANK remains unknown.^6^^,^^7^ Ectonucleotide pyrophosphatase/phosphodiesterase 1 (*Enpp1*; ENPP1) hydrolyzes ATP to PP_i_.^8^^,^^9^ Loss of *Ank* or *Enpp1* results in reduced PP_i_ levels, promoting increased mineralization or ectopic calcification in selective tissues.^3^^,^^9–12^ Developmental studies of *Ank* KO and *Enpp1* KO mice revealed dramatic hypercementosis in both PP_i_-deficient models.^9^^,^^11^^,^^13–15^ Reduction in PP_i_ in these models did not significantly affect the alveolar bone volume or density, showing a remarkable selectivity to cementum.^9^^,^^15^^,^^16^ The periodontal ligament (PDL) space was maintained in the face of rapidly growing cementum via osteoclast-mediated alveolar bone remodeling.^15^^,^^17^^,^^18^

In our previous study, we found that individuals with ENPP1 loss-of-function experienced disrupted tooth eruption and exfoliation as well as difficulties in orthodontic treatment.^19^ Similarly, *Enpp1* KO mice exhibited significantly reduced OTM in the context of increased cementum resorption but decreased dentin resorption.^9^ Studies of *Ank* knock-in mutations (that result in loss of *Ank* function) also revealed reduced tooth movement.^18^ Accumulating evidence suggests that PP_i_ dysregulation impacts periodontal mineral metabolism and tissue remodeling, although the mechanisms remain unclear. We aimed to advance understanding of the role of PP_i_ and increased cementum in tooth movement, root resorption, periodontal remodeling, and their genetic regulators. We used OTM on the *Ank* KO mouse model and analyzed outcomes using high-resolution micro-CT coupled with user-independent 3-dimensional analysis, histology and histomorphometric analyses of root resorption, and a bone-targeted quantitative PCR (qPCR) array to assess gene expression changes.

## Materials and methods

### Animals

Animal experiments were approved (ID: A019-12-06) by the National Institute of Arthritis and Musculoskeletal and Skin Diseases (NIAMS) Animal Care and Use Committee (Bethesda, MD, USA) and conform to ARRIVE (Animal Research: Reporting of In Vivo Experiments) 2.0 guidelines. Mice genetically ablated for the progressive ankylosis gene (*Ank* KO) mice were characterized previously.^5^ Age-matched *Ank* KO and WT control mice were used in experiments (total *n* = 17). Additional details are available in the Supplementary material.

### Orthodontic tooth movement

**Figure 1:**
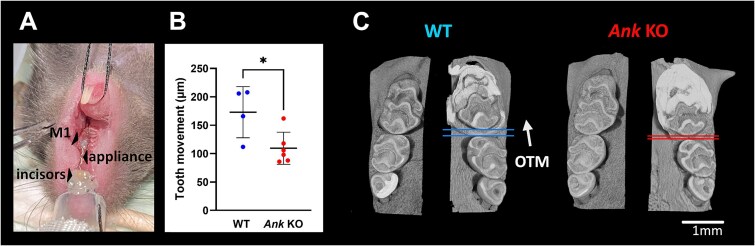
Reduced orthodontic tooth movement (OTM) in the absence of ankylosis protein (ANK). (A) A split-mouth model of OTM was employed using a closed-coil spring stretched between the maxillary first molar and maxillary incisors. The experimental period was 11 days. (B) Compared with WT controls, OTM was significantly reduced in *Ank* KO mice (*n* = 4 and 6, respectively). Results are expressed as mean ± SD. (C) Altered OTM is evident in representative 3D-rendered models of the WT and *Ank* KO mice.

Orthodontic tooth movement was performed as previously described.^9^ Briefly, at 60 days post-natal (dpn), isoflurane-anesthetized WT and *Ank* KO mice (*n* = 4 and 6, respectively, including both males and females) were fixed with an orthodontic appliance. The appliance consisted of a stretched closed-coil spring (0.012-inch nickel-titanium wire; Dentaline GmbH, Germany) ligated between the maxillary left first molar and maxillary incisors using dental flowable composite to apply a force of 0.5 N in the mesial/anterior direction (Figure 1A). In a split-mouth design, the right maxillary first molars were used as contralateral controls (CCs). The split-mouth model was chosen to fulfill the ethical requirements for minimal necessary cohort size and to exclude the interindividual differences.^20^^,^^21^ After the 11-day experimental period, the mice were euthanized with CO_2_.

### Micro-CT analysis

Maxillae were scanned with a μCT 50 scanner (Scanco Medical, Switzerland) at 70 kVp, 76 μA, and 0.5 mm Al filter, achieving an isotropic resolution of 6 μm. The reconstructed images were virtually separated into OTM and CC sides and analyzed using the CTAn software (Bruker Micro-CT, Belgium). Prior to defining the volume of interest (VOI) for analysis, all scans were co-registered in DataViewer (Bruker Micro-CT, Belgium) to a reference consisting of the M1 region (or M2-M3 region for OTM) to ensure the identical position of the VOIs.^22^ Tooth movement was estimated from the same axial plane for all specimens as the smallest distance between M1 and M2 crowns (Figure S1). Additional details of analyses are available in the Supplementary material.

### Histology

Maxillae were fixed in 0.1-M phosphate buffer 4% paraformaldehyde for 24 hours. They were then hemisected, decalcified, and prepared for paraffin histological 2–5-μm serial sagittal sections, which were stained with H&E. Tartrate-resistant acid phosphatase (TRAP) staining was performed to identify osteoclast/odontoclast-like cells. Additional details are provided in Supplementary material.

### qPCR array

Periodontal ligament was harvested on day 3 after OTM initiation to obtain a maximal inflammatory response.^23^ Maxillary first molars from the OTM and CC sides of WT and *Ank* KO mice (*n* = 3 and 4, respectively) were removed from skulls using 15c scalpel blades under a dissecting microscope. Alveolar bone was removed to expose PDL surrounding the root surface of maxillary first molar roots. Care was taken to ensure that teeth were intact to prevent pulpal contamination. Teeth were immediately immersed in MagMAX Lysis/Binding Solution (ThermoFisher Scientific, USA) and vortexed to lyse cells in PDL as well as along root surfaces. Total RNA was isolated, used for transcription of cDNA, and analyzed using a bone-targeted qPCR array (PAMM-170Z, Qiagen, USA). The list of genes analyzed, and additional details are available in the Supplementary material.

### Statistical analysis

**Figure 2:**
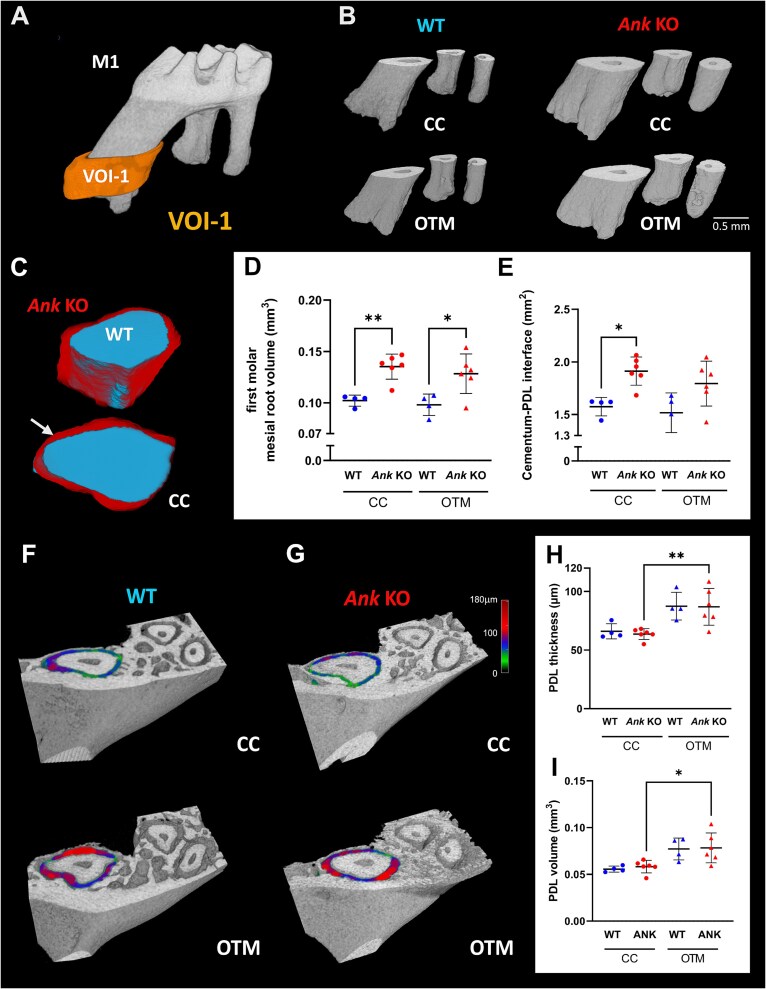
Increased molar root volumes and cementum–PDL interfaces in the absence of ankylosis protein (ANK) while PDL remodeling remains unaffected by genotype. (A) Root and PDL properties were measured using a cylindrical volume (VOI-1) defined around the apical portion of the mesial root of the first maxillary molar (M1). (B) Volume rendering of M1 apical roots reveals larger root volumes in *Ank* KO vs WT mice on both contralateral control (CC) and orthodontic tooth movement (OTM) sides. (C) 3D-registration of WT and *Ank* KO portions of mesial roots of M1 from the CC side reveals increased root circumference in *Ank* mice. Measurements indicate increased M1 (D) root volume and (E) cementum–PDL interface in *Ank* KO vs WT molars. (F, G) Color-mapped images of PDL thickness are color coded to show larger (red) or smaller (green/blue) widths. (H, I) PDL thickness and volume were not affected by genotype but showed trends to significant increases in OTM vs CC sides. Results are presented as mean ± SD. ^*^p < .05; ^**^p < .01.

**Figure 3:**
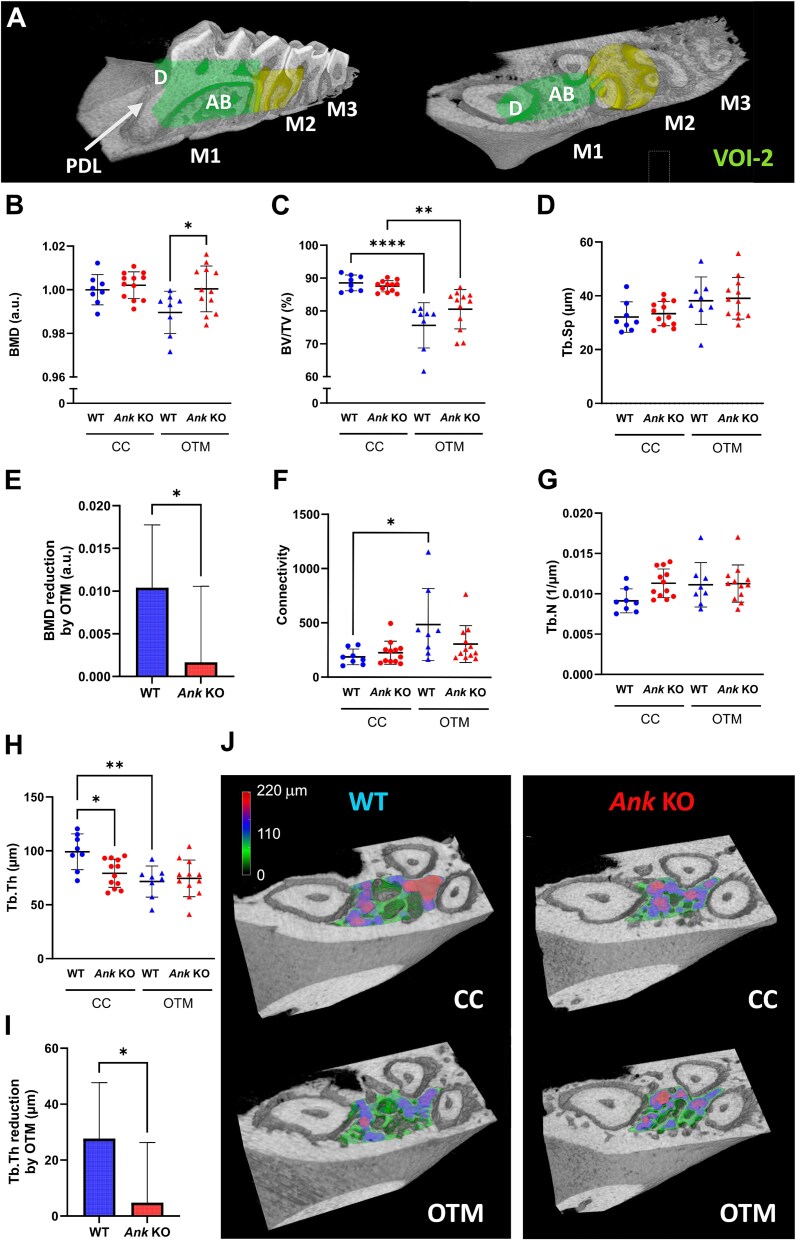
Ankylosis gene (Ank) knockout reduces alveolar bone changes resulting from orthodontic tooth movement (OTM). (A) Volume of interest (VOI-2) used for morphological analysis of alveolar bone within the first molar (M1) trifurcation (highlighted region of M1) and between M1 and second molar (M2) (highlighted region of M2). Dentin (D), alveolar bone (AB), periodontal ligament (PDL), third molar (M3). (B, E) Reduced tissue mineral density (TMD) of AB in WT following OTM was ameliorated in *Ank* KO mice. (C) Reduced bone volume fraction (BV/TV) in both WT and *Ank* KO mice following OTM. (D, G) trabecular separation (Tb.Sp) and number (Tb.N) were comparable between genotypes and were not affected by OTM. (F) Connectivity of trabeculae was significantly increased only in WT mice with OTM; no change was observed in *Ank* KO mice. (H) Trabecular thickness (Tb.Th) of the alveolar bone was significantly reduced in WT mice, while no reduction was detected in *Ank* KO mice. (I) Tb.Th reduction from OTM was smaller in *Ank* KO vs WT mice. (J) A color map representation of Tb.Th within VOI-2 of the M1 socket. Higher density of thicker trabeculae (darker/red) can be seen on the volume-rendered scan of WT on the contralateral control (CC) side. In contrast, thinner (lighter/green) or intermediate (blue) trabeculae predominate after OTM in WT mice. Results are expressed as mean ± SD. ^*^p < .05; ^**^p < .01; ^****^p < .0001.

**Figure 4:**
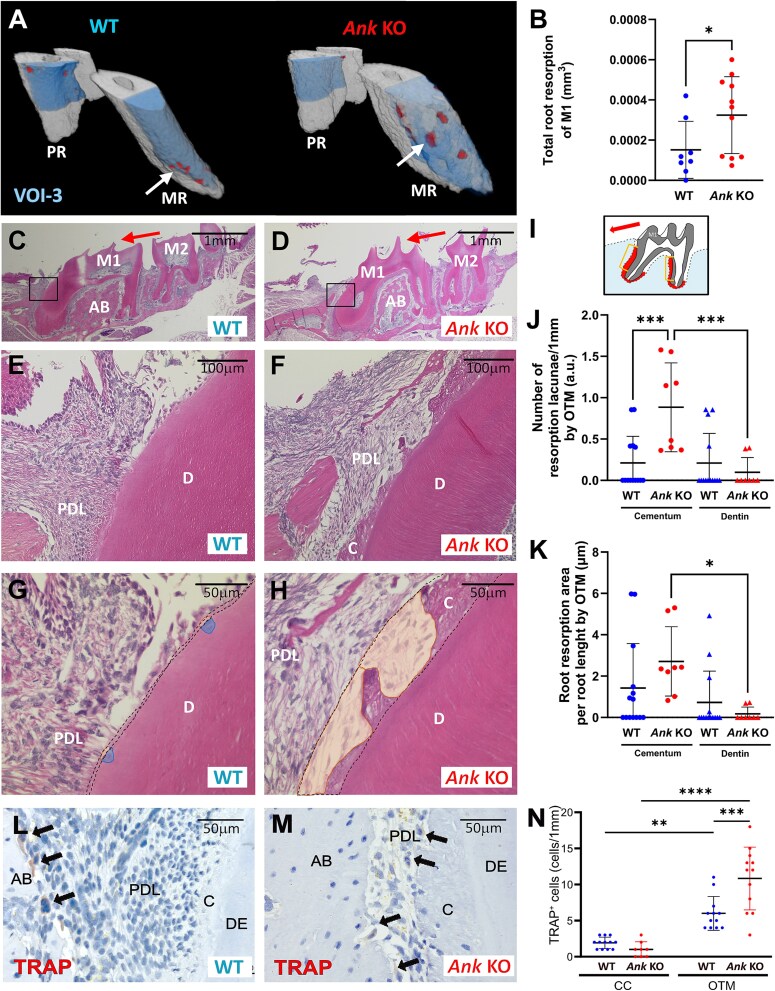
Increased root resorption in the absence of ankylosis protein (ANK). (A) Lateral root resorption (red) was determined by micro-CT from the compression side of the mesial root (MR) and palatal root (PR) of first molar (M1) (highlighted region VOI-3). Arrows indicate root resorption lacunae on the mesial roots. Resorption was increased in *Ank* KO vs WT mice. (B) Root resorption was significantly increased in *Ank* KO vs WT mice. (C–H) Histology of the compression side (boxes in C and D) confirms increased root resorption in *Ank* KO vs WT molars. While comparable areas of resorption were found in dentin (darker/blue areas) and cementum (lighter/yellow areas) in the WT group, the majority of resorption in *Ank* KO mice was found in cementum. Red arrows indicate the direction of orthodontic tooth movement (OTM). Second molar (M2), alveolar bone (AB), dentin (D), periodontal ligament (PDL), cementum (C). (I) Schematic of the lateral area on the compression side of 2 M1 roots (line borders in the red area) considered for measurement of resorption lacunae on histological sections. The arrow indicates the direction of the orthodontic force. (J, K) Measurement of distribution of resorption lacunae observed histologically in both areas, dentin and cementum. (L, M) Focusing on compression zones of M1 distal roots, numbers of TRAP^+^ (tartrate-resistant acid phosphatase) cells (arrows) were counted on OTM and contralateral control (CC) sides. Dentin (DE) (N) Total numbers of TRAP^+^ cells increased after OTM in both genotypes and were greater in *Ank* KO vs WT mice. Results are expressed as mean ± SD. ^*^p < .05; ^**^p < .01; ^***^p < .001; ^****^p < .0001.

All data were analyzed using GraphPad Prism version 9 (GraphPad Software LLC, USA) with α = 5%. Normal distribution was tested by Shapiro-Wilk test. Multigroup comparisons were examined using a 1-way ANOVA with post hoc Tukey test. Orthodontic tooth movement and comparison of OTM-induced changes were evaluated by Student’s *t* test. Statistically significant differences are indicated in the graphs (Figure 2D, E, H, I; Figure 3B-I; Figure 4B, J, K, N) with asterisks according to the corresponding *p* value. All results are presented as mean and SD.

## Results

### Hindered tooth movement in the absence of ANK

Reduced PP_i_ levels and hypercementosis resulting from ENPP1 loss-of-function have been previously found in both mice and humans.^19^ Our previous study established reduced OTM in *Enpp1* KO mice, via undefined mechanisms.^9^ We therefore aimed to investigate whether genetic deletion of ANK, which also reduces PP_i_ levels and promotes hypercementosis, affects OTM in a similar fashion. Our quantitative measurement 11 days after induction of OTM revealed 36% decreased first molar mesial movement in *Ank* KO compared with the WT group (Figure 1B, C).

We next investigated root and PDL structures in relation to OTM. A cylindrically shaped VOI (VOI-1) was defined in the apical part of M1 mesial root (Figure 2A). When comparing *Ank* KO with WT mice, M1 roots were visibly thicker (Figure 2B), as shown after co-registration of CC roots (Figure 2C). As expected, hypercementosis was reflected by a significantly increased tooth root volume in *Ank* KO vs WT mice on both CC and OTM sides (Figure 2D). Cementum–PDL interface was significantly larger in *Ank* KO vs WT mice on the CC side (Figure 2E). Although not statistically significant, this trend persisted on the OTM side.

The PDL surrounding the mesial root in VOI-1 was measured to investigate the effects of genotype and of OTM on the macrostructure of the PDL (Figure 2F, G). The PDL thickness and volume were not affected by *Ank* KO (Figure 2H, I). However, both PDL volume and thickness appeared to increase in association with OTM in both WT and *Ank* KO mice, significantly only in *Ank* KO mice.

### *Ank* KO reduces alveolar bone changes resulting from OTM

A detailed analysis of alveolar bone microarchitecture was performed to understand the effects of *Ank* loss on osteogenesis and bone remineralization during OTM. Morphological analysis within VOI-2 (Figure 3A) on the CC side revealed that alveolar bone parameters were predominantly similar between genotypes. Tissue mineral density, bone volume fraction (BV/TV), trabecular spacing (Tb.Sp), connectivity, and trabecular number (Tb.N) were not different between groups (Figure 3B–G). Trabecular thickness (Tb.Th) was decreased in *Ank* KO vs WT mice (Figure 3H).

A comparison with the OTM side showed significant remodeling differences between WT and *Ank* KO mice. The TMD was more reduced in WT in comparison to *Ank* KO mice due to OTM (Figure 3B, E). BV/TV was reduced in both groups after OTM (Figure 3C). Alveolar bone showed narrower trabeculae already on the CC side of *Ank* KO compared with WT mice. These were not changed as much during OTM (Figure 3H–J).

### Increased cementum resorption in *Ank* KO mice

Reduced OTM in *Ank* KO mice prompted us to measure root resorption and enumerate TRAP^+^ odontoclasts on root surfaces and osteoclasts on alveolar bone surfaces. We used regions of the mesial and palatal roots of the first maxillary molars (VOI-3) (Figure 4A). Micro-CT evaluation revealed a significant increase in total root resorption in *Ank* KO vs WT mice (Figure 4A, B). Distinct resorptive pits were observed along root surfaces of WT and *Ank* KO mice by histology, with larger resorptive areas in *Ank* KO vs WT mice (Figure 4C–H), and with greater amounts of resorption in cementum compared with dentin (Figure 4G–K). While no differences between genotypes were found on the CC side, OTM increased numbers of osteoclast-like cells in both groups, with higher values in *Ank* KO compared with WT mice (Figure 4N).

### Altered gene expression with OTM in the absence of ANK

**Figure 5:**
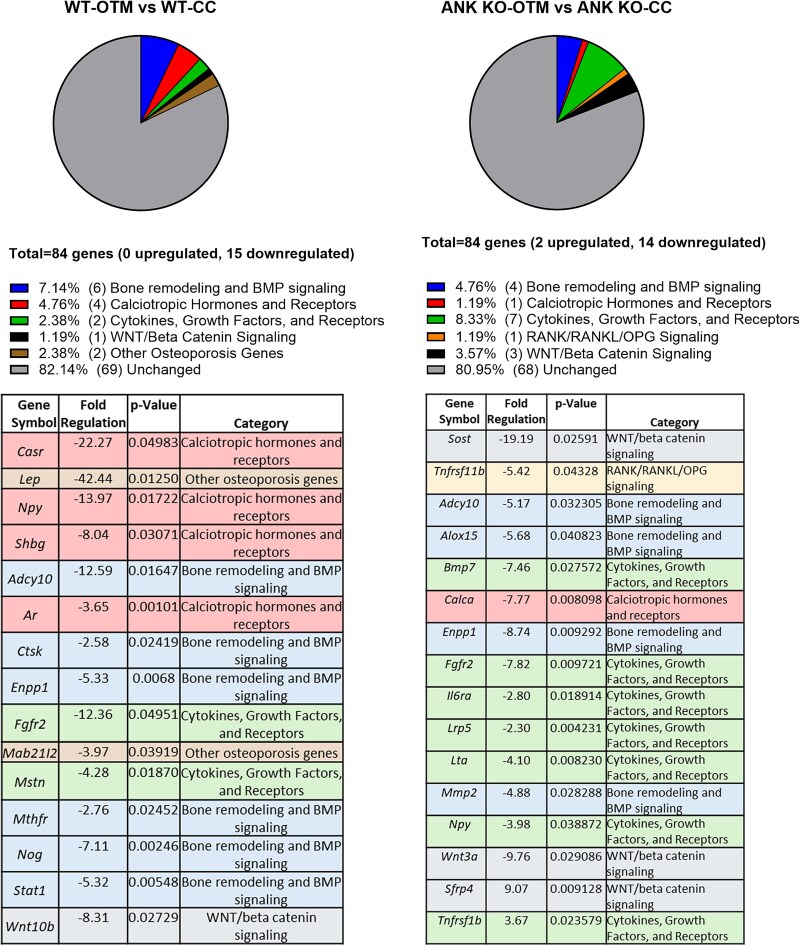
Orthodontic tooth movement (OTM)-induced gene expression changes in periodontal tissues. PDL tissues were carefully removed from the root surface and processed for quantitative PCR (qPCR). qPCR assessment of genes altered with OTM in WT and *Ank* KO mice was performed to help elucidate differences in bone remodeling and root resorption. We selected an array targeted toward bone remodeling to investigate the complex regulation of osteoclastogenesis and mineral metabolism during OTM. A total of 84 genes were assayed, and genes that were altered are categorized in pie charts and listed in tables (unchanged genes are not shown). Percentages and numbers (out of 84) are displayed in the pie charts. Lists show relative expression levels (which were normalized to housekeeping genes) in descending order of fold regulation for genes exhibiting greater than 2-fold change and *p* values < .05. *p* Values and assigned categories are also displayed.

Based on altered OTM and bone remodeling in *Ank* KO vs WT mice, we used a qPCR array for 84 bone-remodeling/turnover-associated markers. RNA was harvested from PDL along tooth root surfaces, and we aimed to identify genes altered from OTM in the presence or absence of *Ank*. Approximately 80% of genes tested were OTM-unaffected in both WT and *Ank* KO mice (Figure 5). Significantly altered genes are categorized into functional groups and listed in Figure 5. In both genotypes, downregulated genes outnumbered upregulated genes.

The bone remodeling and BMP signaling group showed similar changes in WT and *Ank* KO mice with OTM. *Enpp1*, the gene encoding PP_i_ regulator ENPP1, was downregulated in both genotypes. A greater number of calciotropic hormones were significantly reduced in WT vs *Ank* KO mice, including *Casr* (calcium-sensing receptor), *Npy* (neuropeptide Y), *Shbg* (sex hormone–binding globulin), and *Ar* (androgen receptor). *Ank* KO OTM vs CC reduced only *Calca* (calcitonin/calcitonin-related polypeptide alpha) in this functional group. *Ank* KO mice showed greater alteration in cytokines, growth factors, and receptors, with significant reductions in *Bmp7* (bone morphogenetic protein 7), *Fgfr2* (fibroblast growth factor receptor 2), *Il6ra* (interleukin 6 receptor alpha), *Lrp5* (LDL receptor–related protein 5), *Lta* (lymphotoxin alpha), and *Npy*. Notably, *Tnfrsf1b* (TNF receptor superfamily member 1B) is a cytokine that was upregulated in *Ank* KO OTM vs CC mice, but unchanged in WT tissues. Surprisingly, RANK/RANKL/OPG signaling, which coordinates bone resorption, was little changed in either genotype. The notable exception was a more than 5-fold reduction in *Tnfrsf11b* (tumor necrosis factor receptor superfamily member 11b) solely in *Ank* KO mice, better known as osteoprotegerin (OPG), an inhibitor of osteoclast differentiation.

The WNT/beta-catenin signaling group was not the most altered gene set; however, these factors have well-established roles in bone metabolism. *Wnt10b* (Wnt family member 10B), an activator of the WNT signaling cascade, was reduced more than 8-fold in WT tissues. Unlike in WT, *Sost* (sclerostin), a potent inhibitor of Wnt signaling, was reduced more than 19-fold by OTM in *Ank* KO tissues. *Wnt3a* (Wnt family member 3A) was reduced more than 9-fold by OTM in *Ank* KO group. *Sfrp4* (secreted frizzled-related protein 4), another WNT inhibitor and phosphate metabolism regulator, was increased by OTM more than 9-fold in *Ank* KO tissues.

## Discussion

A better understanding of molecular regulators of cementogenesis, as well as the changes in other periodontal tissues during orthodontic tooth movement, is crucial for understanding the orthodontically induced periodontal remodeling process, its side effects, as well as developing therapies to prevent root resorption, bone loss, and PDL detachment. To investigate roles of cementogenesis in PDL and alveolar bone homeostasis, we applied OTM in a split-mouth mouse model, which induces periodontal remodeling, in a hypercementosis model.

Although ANK has roles in PP_i_ metabolism and its loss results in hypercementosis, we did not find altered alveolar bone mineralization due to *Ank* knockout. Similar observations can be found in the literature where *Ank* ablation did not affect mineral densities of alveolar bone,^15^^,^^16^ dentin, enamel,^13^^,^^15^ or cementum.^13–15^ Mechanical properties of enamel, dentin, and cementum were also found to be unaffected.^13^

The large increases in root volume and surface area in *Ank* KO mice compared with WT mice found in this study support the findings of previous studies that discussed the potential of thicker cementum to protect against irreversible tooth root resorption in dentin.^13–15^^,^^17^^,^^19^ Under OTM, we also found an increase in osteoclast/odontoclast cells and increased resorption along root surfaces in *Ank* KO mice. This suggests that OTM increases resorption rate along the tooth root cementum, with *Ank* loss.

The main effect of *Ank* loss on the morphology of the alveolar bone was reduced trabecular thickness (Tr.Th). Alveolar bone in *Ank* KO mice appears to be composed of a somewhat larger number of thinner trabeculae, while the overall mineral density and bone volume remain unchanged. Such findings confirm the results of previous work where alveolar bone volume and mineral density were found to be unchanged in *Ank* KO mice models.^15^^,^^16^ Our work provides the first detailed view of alveolar bone morphology affected by *Ank* loss.

As a key tissue for the mechanical response during orthodontic treatment, the PDL plays an important role in overall dental health. Recently, the functional roles of the PDL has attracted more attention,^22^^,^^24^ but it has not been sufficiently investigated in *Ank* KO mice. Our data confirm the conclusions of previous studies in *Ank* KO mice,^15^^,^^17^ where no effect of *Ank* loss on PDL size, and its remodeling during OTM, was found.

For better clinical orthodontic therapy in patients prone to bone and mineral loss, it is important to understand how mineral metabolism affects bone and PDL remodeling. Several studies have shown how abnormal mineralization can affect tooth movement during orthodontic treatment.^9^^,^^18^^,^^25^^,^^26^ Our study also provides evidence that *Ank* loss leads to significantly impaired tooth movement, comparable to *Enpp1* KO mice, with a parallel mineralization inhibitory effect.^9^ In addition to the potentially altered bone remineralization process, a possible reason may be in the increased root volume, which represents a greater PDL–bone interface, and thus, an additional obstacle to tooth movement. We point out that the split-mouth model may result in an underestimation of OTM due to occlusal compensation and related factors.^20^^,^^21^ Nonetheless, this model is deemed most appropriate for this study as it allows for reduced cohort sizes on ethical grounds, mitigates interindividual variability, and establishes a baseline condition without OTM, with periodontal remodeling being a central focus in both ANK and WT mice cohorts.

Orthodontic tooth movement–induced root resorption may occur by promoting inflammation within the periodontium.^27^ Our study is the first to provide insight into the effect of *Ank* on modulation of tissues and genes within the PDL region associated with OTM-induced root resorption. We demonstrated significantly greater resorption predominantly located in the thicker cementum of *Ank* KO mice. Similar observations were found in *Enpp1* KO mice.^9^ In *Ank* KO mice, thicker cementum may protect against irreversible dentin root resorption. Differences in gene expression and signaling pathways compared with WT mice suggest that mineral metabolism constraints may dictate the cementum thickness or influence the remineralization processes. For instance, the downregulation of *Sost*, a regulator of cementogenesis, during OTM exclusively in *Ank* KO mice might stimulate trabecular production, preventing alveolar bone loss and resulting in more trabeculae compared with WT mice.

Figure 5 shows that calciotropic hormones are significantly downregulated under OTM only in WT mice, indicating that ANK loss may lead to unchanged remineralization processes even under mechanical stress, which may contribute to the slower tooth movement in the *Ank* KO group. Additionally, lower levels of PP_i_, a mineralization inhibitor, in the extracellular matrix of *Ank* KO mice could directly enhance remineralization during OTM.

Based on the expanded cementum width in *Ank* KO and the generally high regenerative capacity of cementum,^9^^,^^28^ we propose that a better understanding of this phenomenon may contribute to the development of therapies against the irreversible root resorption. Our study identified specific genes affected by the loss of *Ank* during OTM, which warrant further investigation as potential therapeutic targets. Understanding ANK’s involvement in cementogenesis may also aid in predicting cases where OTM may be less effective or present an elevated risk of irreversible root resorption. A comprehensive understanding of periodontal phenotypes, related genetic and pathway alterations, and the nature of mechanical stimuli applied will be essential for developing targeted interventions.

Mineralization changes in cementum as well as bone from *Ank* loss likely affect OTM. Whereas *Ank* KO cementum exhibited increased resorption, thinning of trabeculae and TMD reduction of AB were significantly inhibited by loss of *Ank*. Based on our data, the bone remodeling process induced by OTM in *Ank* KO mice is slightly modified. Cementum and bone selectively express similar genes, albeit in different quantities.^29^ Furthermore, unlike bone, cementum is avascular and does not undergo remodeling. These distinctions likely contribute to the different responses of bone vs cementum in OTM.

Downregulation of several osteoclastogenesis-supporting genes (eg, *Casr, Npy, Shbg, Lep*) in WT but not in *Ank* KO mice correlates with the observation of reduced OTM in *Ank* KO mice.^30–32^ One may hypothesize that the suppressed reduction in these mineralization markers in *Ank* KO mice is also associated with the observed hindering of OTM-initiated mineral loss and trabecular thinning. A parallel downregulation of cementogenesis-regulating *Sost* under OTM solely in *Ank* KO mice may contribute to the remineralization effect.^33^  *Sost* KO mice exhibit increased trabecular bone volume in femurs compared with WT mice; *Sost* downregulation in *Ank* KO mice may counteract OTM (and loss of bone on the compression side) by stimulating trabecular production, accounting for the larger number of increased trabeculae (without alveolar bone loss) in *Ank* KO vs WT mice. Downregulation of *Tnfrsf11b* (osteoclast regulator, OPG) in *Ank* KO mice may play a role in the enhanced resorption in cementum, as reported in OPG KO mice.^34^ Further studies on *Ank* gene and other PP_i_ regulators are needed to establish the specific role(s) of PP_i_ modulators in affecting osteoclast, osteoblast, and cementoblast behavior.

*Ank* KO mice showed reduced orthodontic tooth movement, which correlated with reduced effects on alveolar bone, including blunting of bone mineral density loss and thinning of trabeculae resulting from OTM. Counterintuitively, reduced tooth movement coexisted with increased osteoclastogenesis on tooth roots in *Ank* KO vs control mice. Effects on periodontal gene expression may, in part, contribute to this observed discrepancy in osteoclast activity on tooth vs bone surfaces. *Ank* ablation resulted in a dramatic disruption in the gene expression changes associated with OTM. These findings suggest that the *Ank* gene plays a role in directly or indirectly regulating osteoclast/osteoblast activity and bone remodeling in challenge situations like the altered mechanical environment of OTM. Our work provides additional data to recognize *Ank*/ANK/ANKH as a potential target for treatment strategies to modify OTM-induced remodeling or alter root resorption. More work is needed to address key questions such as whether properties of cementoblasts or cementum that normally protect cementum from resorption are subverted by the combination of loss of *Ank* and application of OTM, and whether gene alterations observed here play a part in that shift in clastic activity. Furthermore, can OTM be achieved while simultaneously reducing damaging root resorption? These key questions must be addressed to realize optimal orthodontic therapy in the future.

## Supplementary Material

Revision-Supplementary-Reduced_orthodontic_tooth_ziaf064

## Data Availability

All data for the current study are available from the corresponding author upon reasonable request for academic studies.
