# Supplementary material for: Reduced orthodontic tooth movement in Ank knockout mice
Source: JBMR Plus. 2025 Apr 17;9(6):ziaf064. doi: 10.1093/jbmrpl/ziaf064 (PMC12087959; doi:10.1093/jbmrpl/ziaf064)
Supplement: Revision-Supplementary-Reduced_orthodontic_tooth_ziaf064 [file revision-supplementary-reduced_orthodontic_tooth_ziaf064.docx]

**Reduced orthodontic tooth movement in *Ank* knockout mice**

**Marta Rizk^1*^, Emily Yin Chu^2*^, Rogerio Bastos Craveiro^1^, Merve Elmas^1^, Sihem Brenji^1^, Christian Niederau^1^, Nikolaus Marx^3^, Brian Lee Foster^4^, Martha Joan Somerman^5^, Michael Wolf^1^**

*** contributed equally**

^1^Department of Orthodontics, University Hospital Aachen, Aachen, Germany

^2^Department of Biomaterials and Regenerative Dental Medicine, University of Maryland School of Dentistry, Baltimore, USA

^3^Department of Internal Medicine I - Cardiology, angiology and intensive care medicine, University Hospital RWTH Aachen, Aachen, Germany

^4^Division of Biosciences, College of Dentistry, The Ohio State University, Columbus, USA

^5^National Institute of Arthritis and Musculoskeletal and Skin Disease, National Institutes of Health, Bethesda, USA^+^

^+^study done at NIAMS/NIH, USA (MJS is retired from NIH)

**Supplementary material**

**Materials and Methods**

**Animals**

Animal experiments were approved (ID: A019-12-06) by the National Institute of Arthritis and Musculoskeletal and Skin Diseases (NIAMS) Animal Care and Use Committee (Bethesda, MD, USA). The study conforms to ARRIVE 2.0 guidelines. Mice were fed standard rodent chow, provided access to water *ad libitum*, and maintained on a 12-hour light-dark cycle in a cage of animals. Mice genetically ablated for the progressive ankylosis gene (*Ank* KO) mice were characterized previously (Gurley et al. 2006). Age-matched *Ank^-/-^* (KO) and *Ank^+/+^* wild-type (WT) control mice on a C57BL/6 genetic background were used in the described experiments. The sample size was determined based on the power analysis (G*Power 3.1.9.7, F.Faul, University of Kiel, Germany) and the results of over 30% change in tooth distance after 11 days of OTM in the split-mouth mouse model (Wolf et al. 2018), resulting in a minimum sample size of four. This size was considered as a minimum required size for each group to detect similar effects.

**Micro-computed Tomography**

All maxillae were scanned with a µCT 50 scanner (Scanco Medical, Brüttisellen, Switzerland) at 70 kVp, 76 µA, and 0.5 mm Al filter, achieving an isotropic resolution of 6 µm. The reconstructed images were virtually separated into OTM and CC sides and analyzed. Prior to defining the volume of interest (VOI) for analysis, all scans were co-registered in DataViewer (Bruker Micro-CT, Belgium) to a reference consisting of the M1 region (or M2-M3 region for OTM) to ensure the identical position of the VOIs (Rizk et al. 2023). The registered data were then analyzed using the CTan software (Bruker, Belgium). Tooth movement was estimated from the same axial plane for all specimens as the smallest distance between M1 and M2 crowns (**Suppl. Mat.** **Fig. 1A, B**).


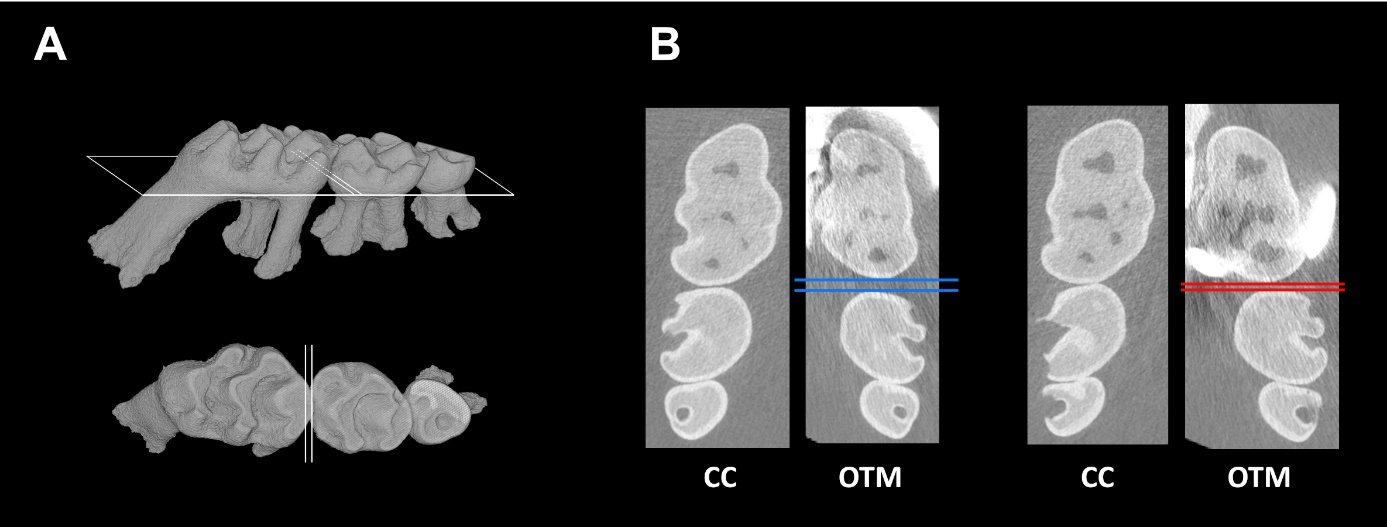


***Ank* KO**

**WT**

**Suppl. Mat. Figure 1.** (**A**) The OTM was estimated from the sections with closest distance between M1 and M2. The identical position of this section was ensured by 3D registration. (**B**) In these sections, the OTM was estimated as the distance between parallel lines drawn at tangents to the teeth surfaces, left/blue (WT), right/red (*Ank* KO).

For the study of tooth roots, a cylindrically shaped VOI-1 (0.81 mm^2^ x 0.36 mm) was defined on the reference scan in the apical part of M1 mesial root (**Fig. 2A**). Roots were isolated from the threshold-based binarized data using a seed method. The volume and surface area of the root were estimated after virtual removal of all pores and pulp to avoid discrepancies in root volume from pulp space variation.

The inner surface of the PDL inside of VOI-1 (**Fig. 2A**) was defined as the outer surface of the root. The outer periphery of the PDL space was modeled after the virtual removal of the root from VOI-1 by a ROI-shrink algorithm with "stretch over pores" option to avoid inclusion of pores in alveolar bone less than 25 voxels, which diffuse up to the PDL. PDL volume and surface were then estimated.

To measure effects of alveolar bone remodeling, two cylindrical regions with an elliptical base were defined within the M1 socket (0.79 mm^2^ x 1 mm), and between M1 and M2 (0.78 mm^2^ x 0.84 mm), defined as VOI-2 (**Fig. 3A**), extending from the crown up to the apical region. Bone and total volume were defined as described previously (Rizk et al. 2023). Large pores attributed to bone marrow or large blood vessels entering the VOI-2 from the apical side were removed. Bone parameters were then estimated: trabecular thickness (Tr.Th) and separation (Tr.Sp); trabecular number (Tr.N); bone volume to total volume ratio (BV/TV); bone mineral density (BMD); trabecular connectivity (Connectivity). The effect of genotype on alveolar bone remodeling was determined by measuring parameter variation (reduction/increase) between the CC and OTM side for each animal.

Lateral root resorption was evaluated in 3D (micro-CT) and 2D (histology, described below) in comparable regions of the mesial and palatal roots of M1. Two regions were defined on the compression sides of the roots, where increased prevalence of root resorption is expected (Trelenberg-Stoll et al. 2021), as VOI-3 (a cuboid covering the cervical root from the cementum enamel junction to approximately mid-root with a length of 0.5 mm (**Fig. 4A**)). Root resorption was determined as the additional volume (> 50 voxels per lacuna) on the root surface after applying the closing algorithm in 3D on the binarized image.

**Histology**

Maxillae were fixed in 0.1 M phosphate buffer 4% paraformaldehyde for 24 hours. They were then hemisected, decalcified, and prepared for paraffin histological 2-5-µm serial sagittal sections, which were stained with hematoxylin and eosin (H&E). Compression zones (**Fig. 4C**) were defined as previously described (Craveiro et al. 2022) on two randomly selected sections per mouse (n ≥4 per group). Comparable areas of resorption lacunae in dentin and cementum were quantified in the WT and *Ank* KO groups.


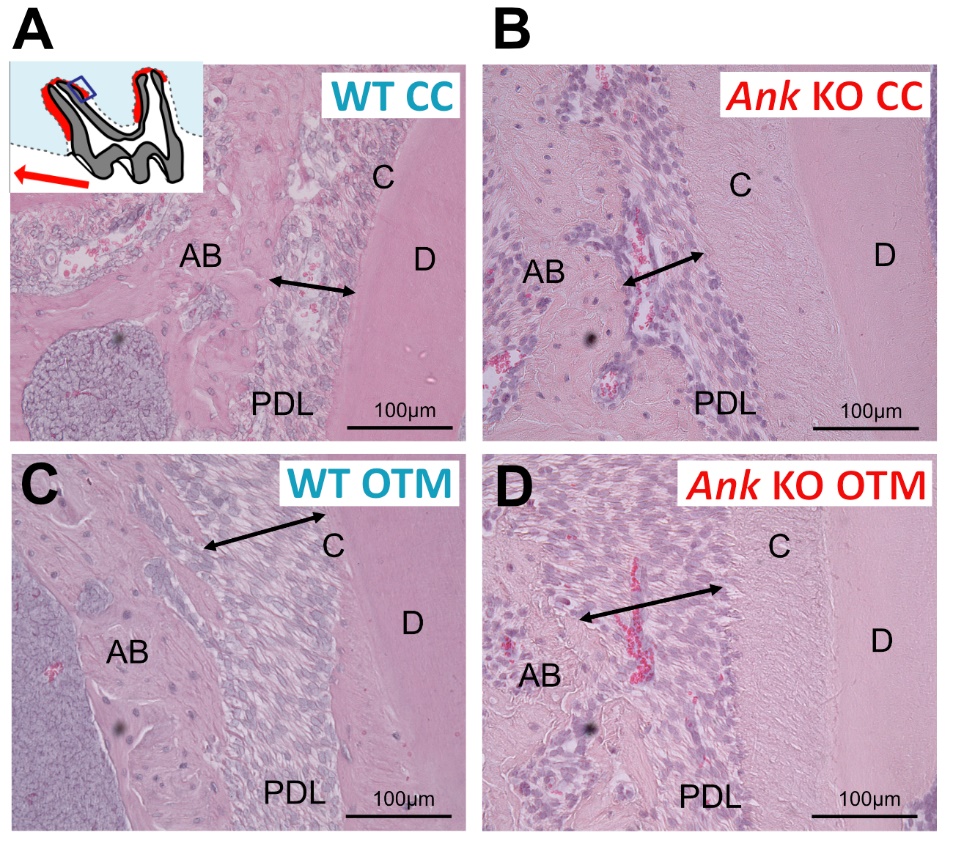


**Suppl. Mat. Figure 2.** Histological observation of the periodontal ligament on the tension side of the apical mesial root (A, blue box) using H&E staining. A relatively small distance between the tooth root and PDL-AB interface (black arrows) was noted in the CC sides of both studied groups: WT (A) and *Ank* KO (B). In contrast, a comparatively larger distance was observed on the OTM side in WT (C) and *Ank* KO (D) mice. Additionally, PDL cells tend to exhibit more unidirectional orientation, with increased disruption of the PDL-AB interface following OTM in both WT and *Ank* KO groups.

TRAP staining was performed to identify osteoclast/odontoclast-like cells. A leukocyte acid phosphatase (TRAP) kit was used according to the manufacturer’s instructions (387A Sigma-Aldrich, Steinheim, Germany). Subsequently, the sections were covered with Aquatex (Merck, Darmstadt, Germany). To quantify the number of TRAP-positive multinucleated osteo/odontoclasts, cells were enumerated in the compression zones (**Fig. 4I**) using Zen software (version 3.3 Zeiss, Jena, Germany).

**Quantitative polymerase chain reaction (qPCR) array**

To investigate genes that were potentially affected by OTM, following removal of maxillary first molars from surrounding bone, PDL was harvested from root surfaces of the maxillary first molar from OTM and contralateral control sides in WT and *Ank* KO mice. Maxillary first molars from the OTM and CC sides were removed from skulls using 15c scalpel blades under a dissecting microscope. Alveolar bone was removed to expose periodontal ligament (PDL) surrounding the root surface. Care was taken to ensure that teeth were intact to prevent pulpal contamination.  Teeth were immediately immersed in MagMAX™ Lysis/Binding Solution (ThermoFisher Scientific, Waltham, MA, USA) and vortexed to lyse cells in PDL as well as along root surfaces. Total RNA was extracted using the MagMAX™ mirVana™ Total RNA Isolation Kit (ThermoFisher Scientific; Waltham, MA, USA) and KingFisher DuoPrime (ThermoFisher Scientific; Waltham, MA, USA).  RNA was used to synthesize cDNA using the RT2 PreAMP cDNA Synthesis Kit (Qiagen; Germantown, MD, USA); subsequently, an osteoporosis qPCR array (PAMM-170Z; Qiagen; Germantown, MD, USA) was employed.  Target gene expression was normalized to 5 housekeeping genes.  PCR array reactions were performed on the LightCycler 480 (Roche Diagnostics, Indianapolis, IN, USA) following manufacturer's recommendations.  Analysis of fold-changes in gene expression was performed using Qiagen’s GeneGlobe Data Analysis Center (https://geneglobe.qiagen.com/us/analyze).  Eighty-four genes were assessed in the array and categorized according to manufacturer’s designations.  Categories assigned were Bone Remodeling and BMP Signaling; Calciotropic Hormones and Receptors; Cytokines, Growth Factors, Receptors; Osteoblast Activity and Differentiation, RANK/RANKL/OPG Signaling, WNT/Beta Catenin Signaling; Other Osteoporosis Genes.

Bone Remodeling & BMP Signaling

Adcy10, Alox12, Alox15, Alox5, Alpl, Bglap, Bmp2, Clcn7, Col1a1, Col1a2, Comt, Crtap, Ctsk, Enpp1, Hsd11b1, Igf1, Itga1, Itgb3, Mmp2, Mthfr, Nfatc1, Nog, Nos3 (eNOS), P2rx7, Plod2, Runx2, Sfrp1, Sost, Sparc, Spp1, Stat1, Timp2, Twist1, Wnt10b, Wnt3a

Calciotropic Hormones & Receptors

Ar, Calca, Calcr, Casr, Cyp17a1, Cyp19a1, Dbp, Esr1 (Erα), Esr2 (Erβ), Esrra, Nr3c1 (GR), Prl, Pth, Pth1r, Pthlh, Shbg, Tshr, Vdr

Cytokines, Growth Factors & Receptors

Bmp2, Bmp7, Cd40, Cnr2, Fgfr1, Fgfr2, Ghrh, Igfbp2, Il15, Il6, Il6ra, Lrp1, Lrp5, Lrp6, Lta (Tnfb), Ltbp2, Mstn, Npy, P3h1, Tgfb1, Tnfaip3, Tnfrsf11a (Rank), Tnfrsf11b (Opg), Tnfrsf1b, Tnfsf11 (Rankl), Vegfa

Osteoblast Activity & Differentiation

Alpl, Bglap, Bmp2, Runx2

RANK / RANKL / OPG Signaling

Tnfrsf11a (Rank), Tnfrsf11b (Opg), Tnfsf11 (Rankl)

WNT / β-Catenin Signaling

Dkk1, Lrp1, Lrp5, Lrp6, Sfrp4, Sost, Twist1, Wnt10b, Wnt3a

Other Osteoporosis Genes

Acp5, Car2, Lep (Leptin), Mab21l2

**References**

Craveiro RB, Florea A, Niederau C, Brenji S, Kiessling F, Sahnoun SEM, Morgenroth A, Mottaghy FM, Wolf M. 2022. [68Ga]Ga-Pentixafor and Sodium [18F]Fluoride PET Can Non-Invasively Identify and Monitor the Dynamics of Orthodontic Tooth Movement in Mouse Model. Cells. 11(19):2949.

Gurley KA, Chen H, Guenther C, Nguyen ET, Rountree RB, Schoor M, Kingsley DM. 2006. Mineral formation in joints caused by complete or joint-specific loss of ANK function. J Bone Miner Res. 21(8):1238–1247.

Rizk M, Niederau C, Florea A, Kiessling F, Morgenroth A, Mottaghy FM, Schneider RK, Wolf M, Craveiro RB. 2023. Periodontal ligament and alveolar bone remodeling during long orthodontic tooth movement analyzed by a novel user-independent 3D-methodology. Sci Rep. 13(1):19919.

Trelenberg-Stoll V, Drescher D, Wolf M, Becker K. 2021. Automated tooth segmentation as an innovative tool to assess 3D-tooth movement and root resorption in rodents. Head Face Med. 17(1):3.
